# The NEDD8-activating enzyme E1 UBA3 orchestrates the immunosuppressive microenvironment in lung adenocarcinoma via the NF-кB pathway

**DOI:** 10.1007/s12032-023-02162-y

**Published:** 2023-09-01

**Authors:** Xiongzhi Lin, Shuhan Yang, Caichuan Zhou, Chengcheng Ao, Dongsheng Sun

**Affiliations:** 1 Taizhou Central Hospital (Taizhou University Hospital), Taizhou University, Taizhou, Zhejiang China; 2 Graduate School of Medicine, Hebei North University, Zhangjiakou, Hebei China; 3 Medical School, Taizhou University, Taizhou, Zhejiang China

**Keywords:** Lung adenocarcinoma, NEDD8-activating enzyme, UBA3, Nuclear factor kappa B, Immunosuppressive microenvironment

## Abstract

Immunosuppressive cells play important roles in generating an immunosuppressive tumor microenvironment and facilitating tumor immune escape. However, the molecular mechanisms underlying their immunosuppressive effects remain unclear. UBA3, the sole catalytic subunit of the neural precursor cell expressed developmentally down-regulated protein 8 (NEDD8)-activating enzyme E1, is highly expressed in various human malignancies, along with an activated neddylation pathway. In this study, we investigated the relationships between the UBA3-dependent neddylation pathway and the infiltration of several immunosuppressive cell populations in lung adenocarcinoma (LUAD). We explored the regulatory mechanisms of UBA3 in LUAD cells by using mRNA sequencing and functional enrichment analyses. Correlations between neddylation and immune infiltrates were assessed by Western blotting, real-time PCR, and analyses of public databases. We found elevated levels of UBA3 expression in LUAD tissues compared to adjacent normal tissues. Blocking UBA3 and the neddylation pathway promoted the accumulation of the phosphorylated nuclear factor of kappa light polypeptide gene enhancer in B-cells inhibitor (p-IκBα), inhibiting the gene expression of tumor cell-derived cytokines such as C–C motif chemokine ligand (CCL) 2, C-X-C motif ligand (CXCL)1, CXCL2, colony-stimulating factor (CSF) 1, CSF2 interleukin (IL)-6, and IL-1B. Moreover, the overexpression of UBA3 in LUAD cells was associated with the secretion of these cytokines, and the recruitment and infiltration of immunosuppressive cells including tumor-associated macrophages (TAMs), plasmacytoid dendritic cells (pDCs), Th2 cells and T-regulatory cells (Tregs). This could facilitate the tumor immune escape and malignant progression of LUAD. Our findings provide new insights into the role of UBA3 in establishing an immunosuppressive tumor microenvironment by modulating nuclear factor kappa B (NF-кB) signaling and the neddylation pathway.

## Introduction

Lung cancer, the leading cause of tumor-related death worldwide [1], can be categorized into small cell lung cancer (SCLC) and non-small cell lung cancer (NSCLC) based on tissue type. NSCLC further includes lung adenocarcinoma (LUAD), lung squamous cell carcinoma (LUSC), and lung large cell carcinoma. LUAD is the most prevalent histological subtype, accounting for 25–30% of all lung cancers [2]. Despite advances in treatments such as surgery, radiotherapy, and chemotherapy over the past decade, the five-year survival rate for lung cancer remains low [3]. Immunotherapy, which utilizes the immune system to clear cancer cells by modulating the immune response, provides a new avenue for the treatment of LUAD [4]. However, only a small subset of patients with cancer can benefit from immunotherapy and the use of immunotherapy as a treatment approach specifically for LUAD faces great challenges [5]. Therefore, it is crucial to clarify the complex interactions between LUAD and the immune system, identify new immunotherapy targets, and provide fresh perspectives for the immunotherapy of LUAD.

In recent years, researchers have been exploring and developing new immunotherapeutic approaches, ensuring the development of this therapeutic area. Among them, immune cell-mediated immune escape has emerged as an important research field across various tumor types [6]. Tumor immune escape is a fundamental characteristic of tumor development, referring to the phenomenon where tumor cells escape recognition and attack from the immune system through various mechanisms. Numerous factors contribute to tumor immune escape, with tumor-induced immunosuppression being the most extensively studied mechanism [7, 8]. Immunosuppressive cells, including tumor-associated macrophages (TAMs), myelogenic suppressor cells (MDSCs) and T-regulatory cells (Tregs), and plasmacytoid dendritic cells (pDCs), play important roles in generating immunosuppressive tumor microenvironment (TME) and facilitating tumor immune escape [9, 10]. For example, TAMs are prone to convert into the M2 immune regulatory phenotype with tumor progression and exert immunosuppressive functions through inhibiting the function of cytotoxic T cells, diminishing the differentiation of Th1 cells, or by recruiting Th2 cells and Tregs [11]. MDSCs within the TME exert immune suppressive activity by inhibiting the function and the proliferation of T cells through cell–cell contact or through release of multiple soluble mediators [12]. Although somewhat controversial, tumor infiltrating pDCs are presumed to play negative roles in some tumors through the induction of Tregs [13, 14]. Targeting immunosuppressive cells in TME has become a burgeoning area of research in tumor immunotherapy research. However, the underlying molecular mechanisms mediating their effects remains incompletely elucidated.

Neddylation is an important post-translational modification of proteins. The small ubiquitin-like molecule neural precursor cell expressed developmentally down-regulated protein 8 (NEDD8) covalently bonds to substrate molecules through the actions of NEDD8-activating enzyme E1, NEDD8- conjugating enzyme E2, and NEDD8 E3 ligase [15, 16]. NEDD8-activating enzyme E1 consists of a heterodimer comprising NAE1 (also known as APPBP1) and UBA3 (also known as NAEβ), NEDD8- conjugating enzyme E2 and NEDD8 E3 ligase mediate the transfer of NEDD8 to the target proteins [17]. The entire neddylation pathway is highly activated, and UBA3 expression level is higher in lung cancer than in adjacent normal tissues and is associated with poor overall patient survival [18]. Moreover, the inhibition of neddylation leads to the accumulation of tumor suppressive substrates such as p21, and WEE1, thus suppressing the proliferation, migration and survival of lung cancer cells [18]. MLN4924, also known as pevonedistat, is a potent and highly selective small molecular inhibitor of UBA3, exhibiting effective antitumor activity in various types of cancer, including lung cancer [19]. To date, over 40 clinical trials have been conducted to evaluate the efficacy of MLN4924, confirming its safety and effectiveness [20].

Currently, many studies focus on the role of UBA3 in the activation of the nuclear factor kappa B (NF-кB) pathway in immunosuppressive microenvironments across different tumors, including nasopharyngeal carcinoma, breast cancer, glioblastoma, retinoblastoma, and liver cancer [21–25]. In our study, we have confirmed that UBA3 may be a major immune checkpoint in tumors. It can influence the immunosuppressive TME and tumor immune escape by regulating the NF-кB pathway and the neddylation process. Consequently, it holds great potential as a new target for tumor therapy.

## Materials and methods

### Bioinformatics analysis

Tumor Immune Estimation Resource (TIMER2.0, http://timer.cistrome.org/), a comprehensive web platform, was used to evaluate the expression of UBA3 and its correlation with immune infiltration [26]. Additionally, the expression of UBA3 across diverse tumor types in the Cancer Genome Atlas (TCGA) was evaluated with the “Gene_DE” module in TIMER2.0. Correlations between UBA3 expression and LUAD-cell-secreted cytokines were determined using the “Gene_corr” module. Moreover, correlations of UBA3 expression with immune cell infiltration in LUAD were investigated using the “Gene” module, after adjusting for tissue purity.

Furthermore, by using UALCAN (http://ualcan.path.uab.edu/) with TCGA datasets [27], we analyzed the gene expression of UBA3 in LUAD or LUSC tissues, comparing it to normal lung controls.

### Cell culture and reagents

Human lung cancer cell lines A549 and H1299 were obtained from American Type Culture Collection (Manassas, VA). Cells were maintained in high glucose DMEM medium (Gibco, CA, USA) with 10% (v/v) fetal bovine serum (Gibco) and 1% (v/v) penicillin–streptomycin solution (Gibco) at 37 °C in a humidified air incubator with 5% CO_2_. MLN4924 was purchased from Selleck Chemicals and dissolved using dimethyl sulfoxide (DMSO, Sigma Aldrich Co., St Louis, MO, USA).

### mRNA sequencing and functional enrichment analyses

H1299 cells were cultured with MLN4924 at a concentration of 1 µmol/L for 24 h. Cells were collected and total RNA was extracted using TRIZOL reagent (Life Technologies, Inc., Waltham, MA, USA) according to the manufacturer’s instruction. The mRNA expression profiles were analyzed by mRNA sequencing (mRNA-seq).

The threshold for significantly differential gene expression was set to a corrected P-value of 0.05. Subsequently, gene ontology (GO)-biological processes (BP) analysis and Kyoto Encyclopedia of Genes and Genomes (KEGG) analysis were conducted to determine the functional enrichments of the differentially expressed genes.

### Western blotting

A549 cells were treated with 1 µmol/L MLN4924 for either 12 h or 24 h. As a control, an equal volume of DMSO was added. Cells from each group were collected, lysed with sodium dodecyl sulfate buffer, and heated at 100 °C for 10 min. Proteins were separated on 10% Bis–Tris polyacrylamide gels by electrophoresis and transferred to polyvinylidene fluoride membranes (Millipore, Bedford, USA). Protein bands were visualized using an enhanced chemiluminescence kit and images were captured using an Amersham Imager 680 (GE Healthcare, IL, USA). Anti-Cullin1 antibody (Abcam, Cambridge, United Kingdom, 75817), anti-phosphorylated nuclear factor of kappa light polypeptide gene enhancer in B-cells inhibitor, alpha (p-IκBα) (Cell Signaling Technology, MA, USA, 2859), anti-IκBα (Cell Signaling Technology, MA, USA, 9242) and anti-β-actin antibody (HuaAn, M1210-2) were purchased.

### RNA isolation and real-time polymerase chain reaction (real-time PCR)

The total RNA was extracted using Trizol reagent (Life Technologies, Inc., Waltham, MA, USA) and treated with RNase-free DNase, following the manufacturer’s instructions. PrimerScript reverse-transcription reagent kit (TaKaRa, Shiga, Japan) was utilized to carry out reverse transcription on 1 µg of total RNA per sample. A real-time PCR assay was performed on the ABI Step One Plus (Applied Biosystems, MA, USA) using the TB Green Premix Ex Taq (TaKaRa). The mRNA abundance in each sample was normalized to the quantity of β-actin. The utilized primer sequences are as follows:

- Human *β-actin*: forward 5′-TGACGTGGACATCCGCAAAG-3′
  reverse 5′-CTGGAAGGTGGACAGCGAGG-3′
- Human *CCL2*: forward 5′- CTCTCGCCTCCAGCATGAAA-3′
  reverse 5′-TTTGCTTGTCCAGGTGGTCC-3′
- Human *CXCL1*: forward 5′-TGCTGCTCCTGCTCCTGGTA-3′
  reverse 5′-AAGCACTGGCAGCGCAGTTC-3′
- Human *CXCL2*: forward 5′-CTGCTCCTGGTGGCCG-3′
  reverse 5′-GCTTCCTCCTTCCTTCTGGT-3′
- Human *IL-6*: forward 5′-ACTCACCTCTTCAGAACGAATTG-3′
  reverse 5′-CCATCTTTGGAAGGTTCAGGTTG-3′
- Human *CSF2*: forward 5′-CCTGGGAGCATGTGAATGCC-3′
  reverse 5′-ATCTGGGTTGCACAGGAAGTT-3′
- Human *IL-1B*: forward 5′-CAGAAGTACCTGAGCTCGCC-3′
  reverse 5′-AGATTCGTAGCTGGATGCCG-3′

### Statistical analysis

The Wilcoxon test was used to evaluate the differential expression of UBA3 in tumor and adjacent normal tissues. We performed a partial Spearman’s correlation analysis to assess the potential correlation between UBA3 expression and immune infiltration. Statistical analysis of the results of real-time PCR was conducted using GraphPad Prism 7. A P value < 0.05 was considered statistically significant.

## Results

### UBA3 RNA expression level is accumulated in LUAD

**Fig. 1:**
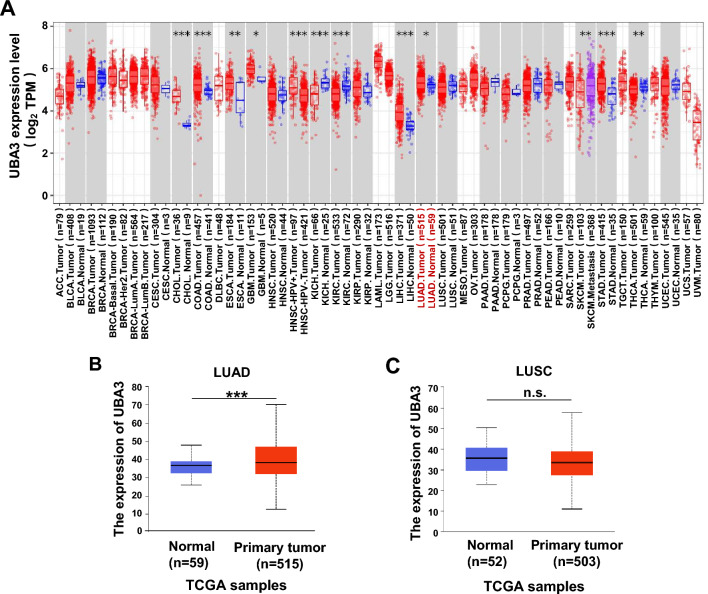
Transcriptional expression of UBA3 in human cancers. **A** UBA3 expression across different cancer types was investigated using the TIMER2.0 database. **B** The level of expression of UBA3 was upregulated in lung adenocarcinoma (LUAD) tissues (n = 515) compared to adjacent normal tissues (n = 59) in samples from the UALCAN database. **C** No significant differences were found in the levels of UBA3 expression between lung squamous cell carcinoma (LUSC) tissues (n = 503) and adjacent normal tissues (n = 52) using data from the UALCAN database (* P < 0.05; ** P < 0.01; ***P < 0.001; n.s., no significant difference)

We explored UBA3 mRNA expression level in tumors from TCGA database by using the TIMER2.0 online database. UBA3 is broadly expressed in various human tumors and normal tissues. Tumor tissues in several types of malignancies showed up-regulated levels of expression of UBA3 compared to adjacent normal controls. These malignancies include cholangiocarcinoma (CHOL), colon adenocarcinoma (COAD), esophageal carcinoma (ESCA), liver hepatocellular carcinoma (LIHC), stomach adenocarcinoma (STAD), and glioblastoma multiforme (GBM). Conversely, the level of expression of UBA3 was down-regulated compared to adjacent normal controls in malignancies such as kidney chromophobe (KICH), kidney renal clear cell carcinoma (KIRC), and thyroid carcinoma (THCA) (Fig. 1A). Notably, in lung cancer, we observed higher level of UBA3 expression in LUAD (n = 515) than adjacent normal tissues (n = 59) (P = 0.0237, Fig. 1A); however, no significant differences were observed between LUSC (n = 501) and the control normal tissues (n = 51) (P = 0.1087, Fig. 1A). We found similar results by analyzing UALCAN database (normal, n = 59 vs. primary LUAD, n = 515; P = 3.4257E-07, Fig. 1B) (normal, n = 52 vs. primary LUSC, n = 503; P = 1.7989E-01, Fig. 1C). The above results show that UBA3 RNA expression level is accumulated in LUAD, which are consistent with previously studies [18]. These findings prompted us to investigate the possible regulatory role of UBA3 in LUAD progression.

### UBA3 is associated with the NF-кB signaling pathway

**Fig. 2:**
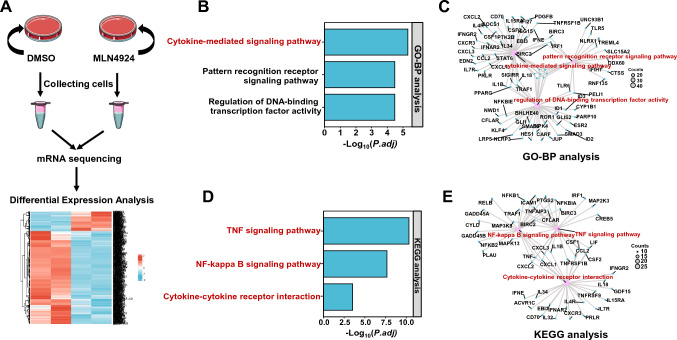
Association of UBA3 with NF-кB signaling pathway. **A** Workflow for RNA sequencing of MLN4924-treated human lung adenocarcinoma A549 cells. **B** Gene ontology (GO)-biological processes (BP) analysis of UBA3-related signaling pathways. **C** GO-BP analysis of main pathway-related cytokines. **D** Kyoto Encyclopedia of Genes and Genomes (KEGG) enrichment analysis of UBA3-related signaling pathways. **E** KEGG enrichment analysis of the main pathway-related cytokines

To explore the regulatory mechanism of UBA3 in LUAD, we analyzed the mRNA expression profiles in A549 lung cancer cells from the solvent-treated and MLN4924-treated groups using mRNA sequencing (mRNA-seq) and conducted enrichment analyses (Fig. 2A). mRNA-seq analysis showed that the treatment with MLN4924 altered the expression of more than 600 genes. We observed the decreased expression of some inflammatory cytokines including C-C motif chemokine ligand (CCL) 2, colony stimulating factor (CSF) 1, CSF2, interleukin (IL)-1B, and C-X-C motif chemokine ligand (CXCL) 1. GO-BP analysis revealed an association of the differentially expressed genes with the cytokine-mediated signaling pathway, as well as the pattern recognition receptor signaling pathway and regulation of DNA-binding transcription factor activity (Fig. 2B and C).

Multiple chemokines and cytokines are significantly enriched in cytokine-mediated signaling pathway in the UBA3 inhibition group (Fig. 2C). KEGG analysis showed that multiple differentially expressed genes were involved in the tumor necrosis factor (TNF) signaling pathway, NF-кB signaling pathway, and cytokine-cytokine receptor interaction (Fig. 2D). Transcription factors and cytokines such as NFKB1/2, RelB, TNF, CCL2, and interleukin (IL)-1B, are involved in these pathways (Fig. 2E). Mounting evidence suggests that NF-кB signaling plays a key regulatory role in multiple cancer types [28–30]. Therefore, we speculate that UBA3 may affect the behaviors of lung cancer cells and contribute to cancer-associated inflammation by regulating the NF-кB pathway.

### UBA3-dependent neddylation pathway regulates the transcriptional activation of tumor-related factors via the NF-кB signaling pathway

**Fig. 3:**
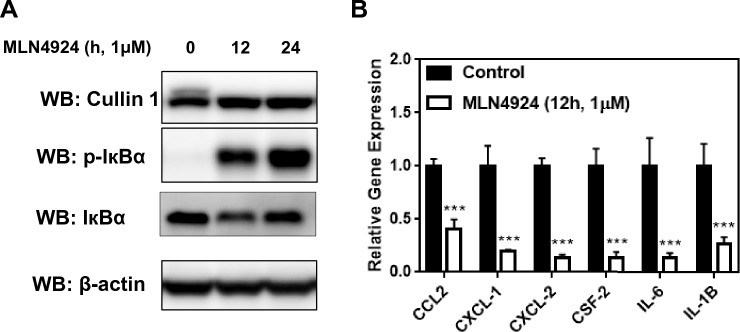
UBA3-dependent neddylation pathway has effect on the transcriptional activation of tumor-related cytokines mediated by the NF-κB signaling pathway. **A** MLN4924 treatment led to the accumulation of phosphorylated IκBα. Protein expression of Cullin1, p-IκBα, IκBα and β-actin in A549 cell lysates were detected by Western blot. **B** The level of expression of certain tumor-related cytokines was decreased in MLN4924-treated A549 groups. Relative mRNA levels of expression of CCL2, CXCL1, CXCL2, CSF2, IL-6, and IL-1B were evaluated by real-time PCR. β-actin was used as an internal reference. (* P < 0.05; ** P < 0.01; ***P < 0.001)

To verify whether the inhibition of UBA3-dependent neddylation could affect NF-кB signaling, we analyzed the expression of related key proteins in MLN4924-treated A549 cells. As shown in Fig. 3A, MLN4924 treatment affected the neddylation of Cullin1, a crucial cullin component, and a substrate of NEDD8 E3 ligases. Moreover, we found that UBA3 inhibition promoted the accumulation of p-IκBα in a time-dependent manner (Fig. 3A). These results support the idea that the inhibition of the neddylation pathway leads to accumulation of p-IκBα and the blockage of NF-кB translocation [31].

**Fig. 4:**
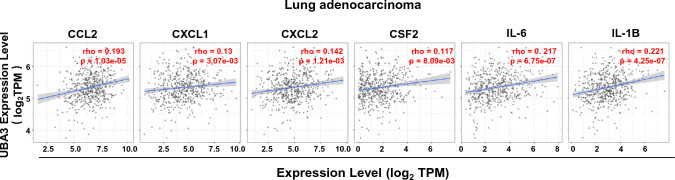
Correlation between the levels of expression of UBA3 and cytokines. The level of expression of UBA3 was positively correlated with that of cytokines including CCL2, CXCL1, CXCL2, CSF2, IL-6, and IL1-B in lung adenocarcinoma cells using data from the TIMER2.0 database

Next, we validated the gene expression of several potential chemokines and cytokines related to the NF-кB signaling pathway and immunosuppressive cell infiltration. The real-time PCR results showed that MLN4924-treated A549 cells had significantly lower levels of expression of genes including CCL2, CXCL1, CXCL2, CSF2, IL-6, and IL-1B than control cells (all P < 0.001, Fig. 3B). Additionally, publicly available data also indicate significant positive correlations between the expression of UBA3 and these factors in TCGA-LUAD datasets (CCL2: rho = 0.193, P = 1.03E-05; CXCL1: rho = 0.13, P = 3.07E-03; CXCL2: rho = 0.142, P = 1.21E-03; CSF2: rho = 0.117, P = 8.09E-03; IL-6: rho = 0.217, P = 6.75E-07; IL-1B: rho = 0.221, P = 4.25E-07) (Fig. 4). These results suggest that blocking UBA3 and subsequent neddylation pathway could inhibit LAUD-cell-secreted chemokines and cytokines, possibly by suppressing NF-кB signaling pathway.

### The expression of UBA3 is associated with immune cell infiltration in LUAD

**Fig. 5:**
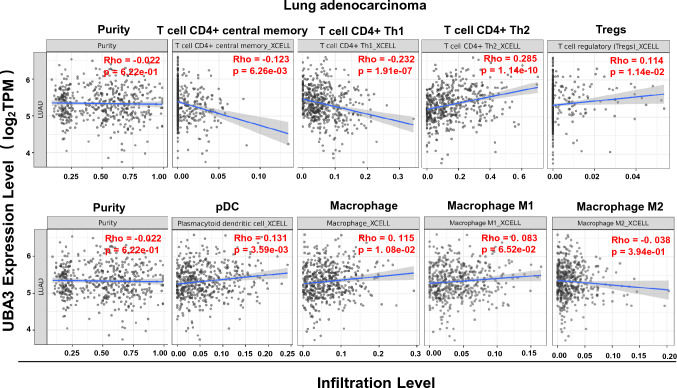
Correlation between the level of expression of UBA3 and immune cell infiltration. The level of expression of UBA3 is positively associated with immune cell infiltration including Th2 cells, Tregs, pDCs, and TAMs in lung adenocarcinoma cells using data from the TIMER2.0 database

Tumor-secreted factors contribute to the infiltration of immune cells, facilitating an immunosuppressive TME and leading to immune cell-mediated immune escape. Therefore, we assessed the association between UBA3 expression and several major types of immune suppressive infiltrates using the TIMER2.0 database. As shown in Fig. 5, UBA3 expression is significantly negatively correlated with the infiltration levels of Th1 cells (rho = − 0.232, P = 1.91E-07); however, it is positively correlated with immune suppressive cells, including Th2 cells (rho = − 0.285, P = 1.14E-10), Tregs (rho = 0.114, P = 1.14E-02), pDCs (rho = 0.131, P = 3.59E-03), and TAMs (rho = 0.115, P = 1.08E-02) (Fig. 5). These data indicate that the overexpression of UBA3 in LUAD cells may promote the recruitment and infiltration of immunosuppressive cells by secreting chemokines and cytokines such as CCL2, CXCL1, and CSF2 via NF-кB signaling.

## Discussion

**Fig. 6:**
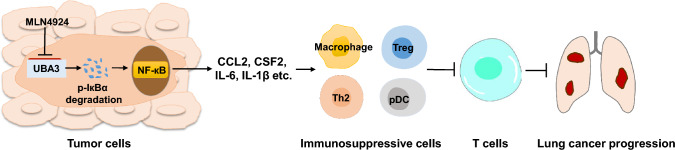
Working model illustrating the regulatory role of UBA3 in lung adenocarcinoma cells for promoting immune suppressive infiltrates

Tumor cells have the potential to recruit immune cells and establish an immunosuppressive TME, aiding tumor cells to evade immunological surveillance and facilitating malignant progression [32]. LUAD, the most common subtype of primary lung cancer and one of the most fatal malignancies worldwide poses challenges for current treatment approaches, including immunotherapy. Understanding the immune-modulatory role of tumor cells and the complex mechanisms of immune evasion in LUAD is crucial. Herein, we found that UBA3 expression was elevated in LUAD tissues. Blocking UBA3 and the subsequent inhibition of neddylation pathway promoted the accumulation of p-IκBα and suppressed the expression of LAUD-cell-derived chemokines and cytokines, including CCL2, CXCL1, CXCL2, CSF2, IL-6, and IL-1B, possibly through the NF-кB signaling pathway. Moreover, the overexpression of UBA3 in LUAD cells may promote the recruitment and infiltration of immunosuppressive cells such as Th2 cells, Tregs, pDCs, and TAMs by secreting the above-mentioned cytokines, and facilitate the tumor immune escape and malignant progression in LUAD (Fig. 6). Our findings provide new insights into the role of UBA3 in the development of an immunosuppressive TME by regulating the neddylation pathway and influencing NF-кB signaling interaction.

As the sole catalytic subunit of NAE1, UBA3 is essential for activating NEDD8 and facilitating the neddylation process [33]. Aberrantly high expression of UBA3 has been implicated in a wide range of human malignancies and is usually associated with tumor progression through modulation of the properties of tumor cells [34]. Furthermore, UBA3 and other neddylation enzymes are overexpressed in lung cancer tissues compared to adjacent normal tissues. Blocking the neddylation using MLN4924 significantly inhibited the malignant phenotype of LUAD cells, including proliferation, clonogenic survival, and migration [18]. Additionally, the abrogation of the neddylation pathway has also demonstrated chemosensitizing effects in LUAD cells [18]. Consistent with these findings, using data from the publically available databases, our study reveals higher levels of gene expression of UBA3 in LUAD tissues than in adjacent normal tissues.

Neddylation not only directly targets lung cancer cells, but also influences the TME. Inactivation of the neddylation pathway inhibits the activity of NEDD8 E3 ligase, leading to the accumulation of phosphorylated IкBα and blocking NF-кB translocation [18, 34]. This inhibition reduces CCL2 secretion in lung cancer cells and decreases the infiltration of monocytes/TAMs [35]. We recently showed that targeting the neddylation pathway with MLN4924 inhibits NF-κB signaling and suppresses mCXCL5-mediated MDSC infiltration in a Lewis lung cancer model [31]. Consistent with these findings, we confirmed the involvement of the neddylation pathway in NF-κB-mediated tumor immunity. MLN4924 treatment disrupted NF-кB signaling by affecting the neddylation of cullin1 and promoting the accumulation of p-IκBα in LUAD cells. Furthermore, our mRNA sequencing analysis detected a decreased expression of several related cytokines, including CCL2, CXCL1, and CSF2 in the MLN4924-treated group. These tumor-cell-secreted factors have been linked to the recruitment or activation of several immunosuppressive populations of immune cells. Immunosuppressive cells play crucial regulatory roles in the progression of lung cancer, and the feedback between tumor and immunosuppressive cells promotes tumor progression. For example, tumor-cell-secreted factors like CCL2 and CSF1, contribute to the recruitment and survival of TAMs, which in turn promote immune evasion, cancer proliferation, epithelial-mesenchymal transition, and tumor invasiveness in lung cancer [35]. This prompted us to explore the association between UBA3 and immune suppressive cell infiltration. We found that UBA3 expression is positively correlated to the infiltration levels of Th2 cells, Tregs, pDCs, and TAMs, suggesting its potential regulatory role in the formation of an immunosuppressive microenvironment.

TAMs are the predominant immune cell population with the TME. In general, TAMs are considered to switch their phenotypes from the classical M1 to the alternative M2 and exhibit immunosuppressive properties as the tumor advances [36, 37]. In the present study, UBA3 expression level is positively correlated with the infiltration level of TAMs. However, analysis of UBA3 expression in TCGA-LUAD tumors and adjacent normal tissues shows no significant association with and M1 and M2 TAM subtypes by using the analytical XCELL algorithm. This may link to the phenotypic plasticity of macrophages in vivo and different stages of LUAD [38]. Evidence suggests that tumor derived chemokines and cytokines including CCL2, CSF1, ECM components and hypoxia are involved in the recruitment of monocytes/macrophages [39, 40]. Our present work shows MLN4924-treated lung cancer cells had lower gene expression of CCL2 and CSF1 (data not shown) than controls, suggesting that CCL2, CSF1 may participate in modulating the TAM infiltration in LUAD. Tregs are a crucial therapeutic target in the TME, they can be recruited to the TME by macrophages and tumor-derived factors such as CXCL12, CCL17 CCL22 and CCL1, thus suppressing the activation of T cells [41]. However, our mRNA-seq data show no differential expression of the above factors in the lung cancer cells. We speculated that TAMs may play an indirect role in the recruitment of Tregs and Th2 cells. In addition, public data analysis indicates that UBA3 expression is also positively correlated with the infiltration of pDCs, which are generally presumed to play negative roles through the induction of Tregs [14, 42]. Complex factors including CCL2 may be involved [43].

Some limitations of this study must be stated. First, the correlations between UBA3 and immune suppressive infiltrates need further verification through in vitro experiments or tumor‐bearing animal models. Second, the underlying mechanisms by which UBA3 regulates the immunosuppressive TME require further intensive investigation.

In summary, this study provides evidence that high levels of expression of UBA3 in LUAD may promote the infiltration of Th2 cells, Tregs, pDCs, and TAMs by promoting various tumor-derived factors, thus facilitating the progression of lung cancer. UBA3 may be a major immune checkpoint and serve as a potential target for immunotherapy in lung cancer.
